# N-Chlorotaurine, a Long-Lived Oxidant Produced by Human Leukocytes, Inactivates Shiga Toxin of Enterohemorrhagic *Escherichia coli*

**DOI:** 10.1371/journal.pone.0047105

**Published:** 2012-11-06

**Authors:** Christian Eitzinger, Silvia Ehrlenbach, Herbert Lindner, Leopold Kremser, Waldemar Gottardi, Dmitri Debabov, Mark Anderson, Markus Nagl, Dorothea Orth

**Affiliations:** 1 Department of Hygiene, Microbiology and Social Medicine, Division of Hygiene and Medical Microbiology, Innsbruck Medical University, Innsbruck, Austria; 2 Division of Clinical Biochemistry, Biocenter, Innsbruck Medical University, Innsbruck, Austria; 3 NovaBay Pharmaceuticals, Inc., Emeryville, California, United States of America; University of Osnabrueck, Germany

## Abstract

*N*-chlorotaurine (NCT), the main representative of long-lived oxidants produced by granulocytes and monocytes, is known to exert broad-spectrum microbicidal activity. Here we show that NCT directly inactivates Shiga toxin 2 (Stx2), used as a model toxin secreted by enterohemorrhagic *Escherichia coli* (EHEC). Bacterial growth and Stx2 production were both inhibited by 2 mM NCT. The cytotoxic effect of Stx2 on Vero cells was removed by ≥5.5 mM NCT. Confocal microscopy and FACS analyses showed that the binding of Stx2 to human kidney glomerular endothelial cells was inhibited, and no NCT-treated Stx2 entered the cytosol. Mass spectrometry displayed oxidation of thio groups and aromatic amino acids of Stx2 by NCT. Therefore, long-lived oxidants may act as powerful tools of innate immunity against soluble virulence factors of pathogens. Moreover, inactivation of virulence factors may contribute to therapeutic success of NCT and novel analogs, which are in development as topical antiinfectives.

## Introduction

*N*-chlorotaurine (Cl-HN-CH_2_-CH_2_-SO_3_H, NCT), an *N*-chloro derivate of the amino acid taurine, is a long lived oxidant produced in high amounts by stimulated human granulocytes and monocytes [1]–[3]. It has been shown to downregulate in vitro proinflammatory chemokines, cytokines, and enzymes, such as TNFα, nitric oxide, and interleukins 1beta, 2, 6, 8, 12 [4]–[6]. Oxidation of IκBα and the following inhibition of activation of NF-κB is assumed as a major basic mechanism [7], as well as induction of hemoxygenase-1 [8].

Furthermore, NCT exerts broad-spectrum microbicidal activity down to micromolar concentrations, so that its contribution to inactivation of pathogens by phagocytes has been discussed [9], [10]. The possibility to synthesize the substance chemically as crystalline sodium salt (Cl-HN-CH_2_-CH_2_-SO_3_-Na, [11]) paved the way for numerous studies investigating mainly the 55 mM (1% w/v) aqueous solution on its suitability as a topical antiinfective agent in human medicine (for review see [12]). One major advantage is the very good tolerability as a mild endogenous active chlorine compound, so that sensitive body regions and cavities can be irrigated [12]. These findings encouraged the development of analog chloramines with even higher solution stability, *N*-monochloro-dimethyltaurine (Cl-HN-CH_2_-(CH_3_)_2_-CH_2_-SO_3_-Na, NVC-612) and *N,N*-dichloro-dimethyltaurine (Cl_2_-N-CH_2_-(CH_3_)_2_-CH_2_-SO_3_-Na, NVC-422), which are promising new antiinfective agents [13]–[15].

Investigating the mechanisms of action of NCT, we found that chlorination of the surface of bacteria and fungi occurred within 1 min, before the killing started [16]. Importantly, incubation of bacteria for such sublethal times in NCT removed their virulence, as demonstrated for *S. aureus* and *S. pyogenes* in the mouse peritonitis model [9], [17]. Moreover, secretory aspartyl proteinases of *Candida albicans* were found to be downregulated by sublethal concentrations of NCT [18]. Loss of virulence was connected with a lag of regrowth of pathogens, generally designated as postantibiotic effect [9], [17], [18].

Besides that, we hypothesized that not only the pathogens can be attacked by NCT, but also their virulence factors may be directly oxidized and inactivated. This concept was supported by the finding that gliotoxin of *Aspergillus fumigatus* is obviously inactivated by this chlorine compound [19]. The consequences would be at least dual: First, long-lived oxidants produced by granulocytes and monocytes may act as tools of innate immunity to inactivate secreted or surface-bound virulence factors. Second, upon clinical application of chloramines as antiinfective solutions, an impact on the metabolites of pathogens in addition to the microbicidal effect could enhance the therapeutic success rate.

To address particularly the first issue, we decided to investigate in the present study in detail the influence of NCT, NVC-422 and NVC-612 on a clinically important secreted bacterial toxin, which causes granulocyte invasion into the tissue. We chose Shiga toxin 2 (Stx2), which is produced by enterohemorrhagic *Escherichia coli* (EHEC) [20]. Stx2 consists of an enzymatically active A subunit (32 kDa) and a non-covalently linked B subunit pentamer (7,7 kDa for each monomer) responsible for interaction with glycolipid receptors on target eukaryotic cells. The A subunit possesses N-glycosidase activity and cleaves a single adenine residue from 28S ribosomal RNA. This depurination results in inhibition of protein synthesis and target cell death. The amino acid sequence of Stxs has already been determined [21], which was important for the performance of our present study.

EHEC are the major cause of hemolytic-uremic syndrome (HUS) in childhood. HUS is characterized by a clinical triad of microvascular glomerular thrombosis, consumptive thrombocytopenia and microangiopathic hemolytic anemia. The thrombotic microangiopathy is especially severe in the kidney and can lead to acute renal failure. This is connected with activation of the complement system [22], with infiltration of neutrophilic granulocytes in the glomeruli [23], [24] and with leukocytosis in peripheral blood [25]. It may be hypothesized that one effect of leukocytes is inactivation of the causative agent of microangiopathy and inflammation, i.e. Shiga toxin. Long-lived oxidants could be involved in this process.

The aim of this study was to investigate for the first time the impact of NCT on a virulence factor from the molecular process to the functional and biological consequences. Stx2 was used as a model for an important secreted toxin, which causes infiltration of NCT-producing leukocytes.

## Results

### Inhibition of Growth of EHEC and of Stx2 Production in the Presence of Sublethal Concentrations of NCT

**Figure 1:**
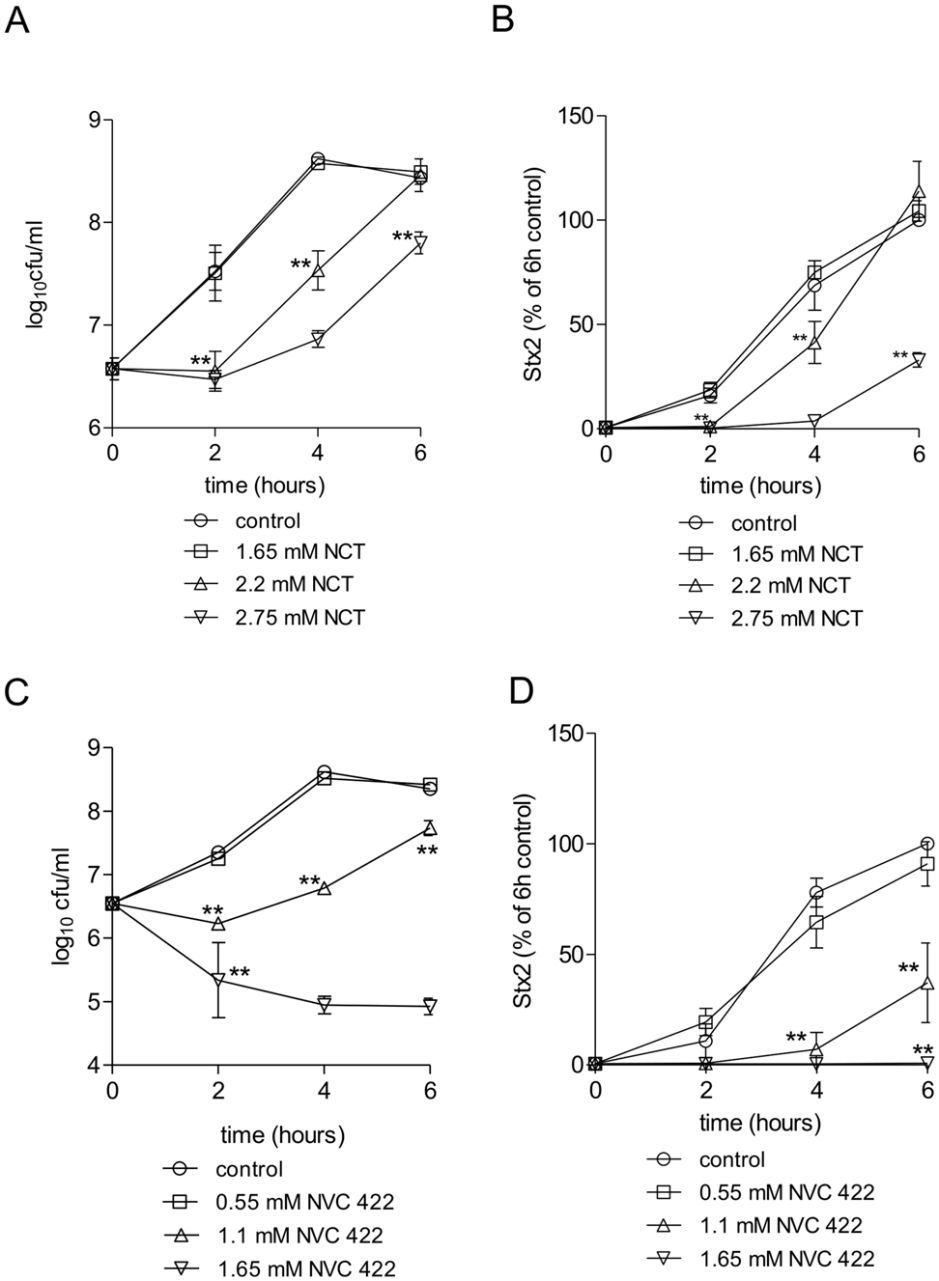
Inhibition of growth of EHEC 178 and of Stx2 production in the presence of sublethal concentrations of NCT and NVC-422. (A) CFU counts of EHEC in Direct Medium, to which NCT was added to final concentrations of 0 mM (control), 1.65 mM, 2.2 mM and 2.75 mM at 37°C. (B) Stx2 produced by EHEC under the same conditions as in (A), measured by ELISA, related to the 6 h value of the control without NCT. (C) CFU counts of EHEC in Direct Medium, to which NVC-422 was added to final concentrations of 0 mM (control), 0.55 mM, 1.1 mM and 1.65 mM at 37°C. (D) Stx2 produced by EHEC under the same conditions as in (C), measured by ELISA, related to the 6 h value of the control without NVC-422. Mean values ± SD from three independent experiments are shown in (A–D). *P<0.05; **P<0.01.

The CFU counts of EHEC 178 in the presence of 1.65 mM, 2.2 mM and 2.75 mM NCT in EHEC Direct Medium are shown in Figure 1A and the Stx2 production in Figure 1B (Figure S1A and B for EHEC 22). Bacterial growth from the starting point of 6.5 log_10_ was necessary for detection of Stx2. NCT (1.65 mM) had no influence on both bacterial growth and toxin production. For 2.2 mM and 2.75 mM, a growth inhibition of 2 and 4 h was observed, respectively. The course of Stx2 levels was similar, and the toxin production was blocked for 2 and 4 h by 2.2 mM and 2.75 mM NCT. Very similar results were found for 1.65–2.75 mM NVC-612 (data not shown). Compared to NCT and NVC-612, NVC-422 exhibited stronger activity (Figure 1C and D and Figure S1C and D for EHEC 22). Already 1.65 mM NVC-422 led to a reduction of the bacterial count by approximately 1.5 log10 and completely abrogated Stx2 production. A lag of regrowth and inhibition of Stx2 for 4 h was caused by 1.1 mM NVC-422, while 0.55 mM had no effect on both parameters. This can most likely be attributed to the higher oxidation capacity because of the two chlorine atoms in the molecule.

The tests with EHEC 22 confirmed all these results with a lag time of 2–4 h for the same concentrations of test substances and for both parameters (Figure S1A–D).

### Inhibition of the Cytotoxic Effect of Stx2 against Vero Cells by NCT

**Figure 2:**
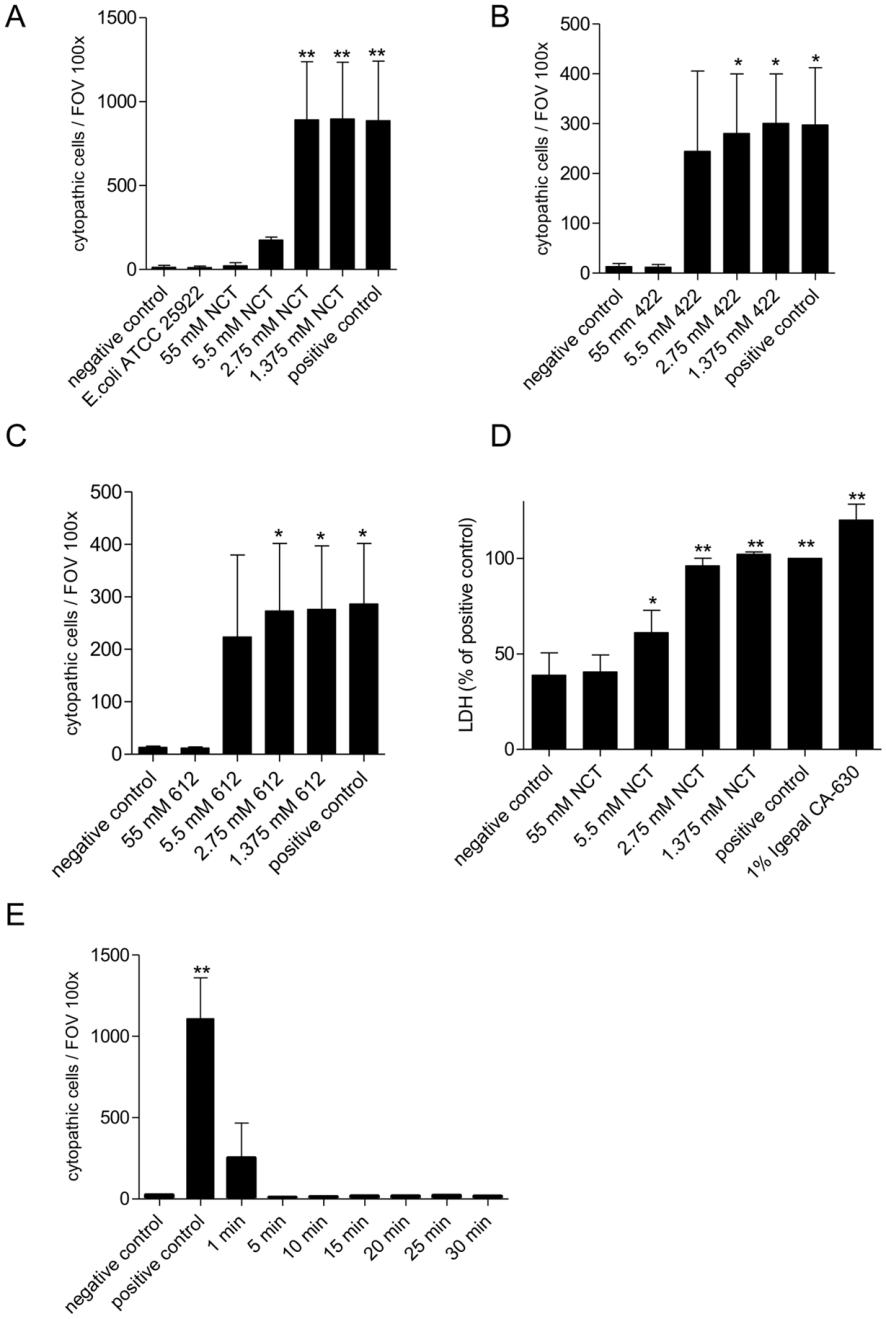
NCT, NVC-422, and NVC-612 inhibit the cytopathic effect of Stx2 in Vero cells. Supernatants of EHEC 178 cultures were treated with 1.38–55 mM of oxidants for 30 min (A–D), diluted 100-fold in RPMI + 10% v/v FCS, and added to Vero cell cultures, which were monitored for the typical cytopathic effect. (A–C) Numbers of cytopathic cells per visual field at a 100x magnification after incubation with EHEC supernatant for 72 h. Comparison of RPMI + FCS without Stx (negative control), supernatant of the non-EHEC strain ATCC 11229 (shown in A), untreated supernatant (positive control), and supernatant treated with NCT (A), NVC-422 (B), or NVC-612 (C). (D) Quantification of cell death by LDH assay after incubation with EHEC supernatant. Values were related to untreated supernatant. An additional separate positive control was performed with 1% Igepal. (E) Supernatants of EHEC 178 cultures were treated with 55 mM NCT for 1–30 min, followed by Vero cell assay and evaluation as in A–C. Mean values ± SD from three independent experiments are shown in (A–E). *P<0.05; **P<0.01.

To investigate the capability of NCT and its analogs to impact the biological function of Stx2, the supernatant of EHEC cultures was treated for 30 min with the oxidants and added to Vero cells. Cells were monitored on the typical cytopathic effect by light microscopy for 3 days (Figure 2). When 55 mM (final concentration) NCT was added to the supernatant, only few dead cells (<5%) were seen as in controls treated with RPMI + FCS without any additives. A cytopathic effect typical for Stx was completely absent. With 5.5 mM NCT, some cells (approximately 20% of the value of the positive control) showing the Stx-specific cytopathic effect could be seen, although the total number of cytopathic cells was not significantly higher compared to the untreated control (Figure 2A). The results with NVC-422 and NVC-612 were very similar (Figure 2B and 2C). LDH assay (Figure 2D) and trypan blue exclusion tests (data not shown) confirmed these findings. A kinetic experiment disclosed that the inhibition of the Stx effect by NCT occurred already after 1 min (Figure 2E). The results were the same for both EHEC strains (data not shown for strain no. 22). In contrast to growth inhibition of EHEC (see above), NVC-422 offered the same activity than the other two test compounds. Taurine and dimethyltaurine (55 mM each) added to the supernatant of EHEC cultures did not decrease the number of cells showing a cytopathic effect and therefore did not influence the activity of Stx2 (data not shown).

### Consumption of Oxidation Capacity by EHEC Medium

**Figure 3:**
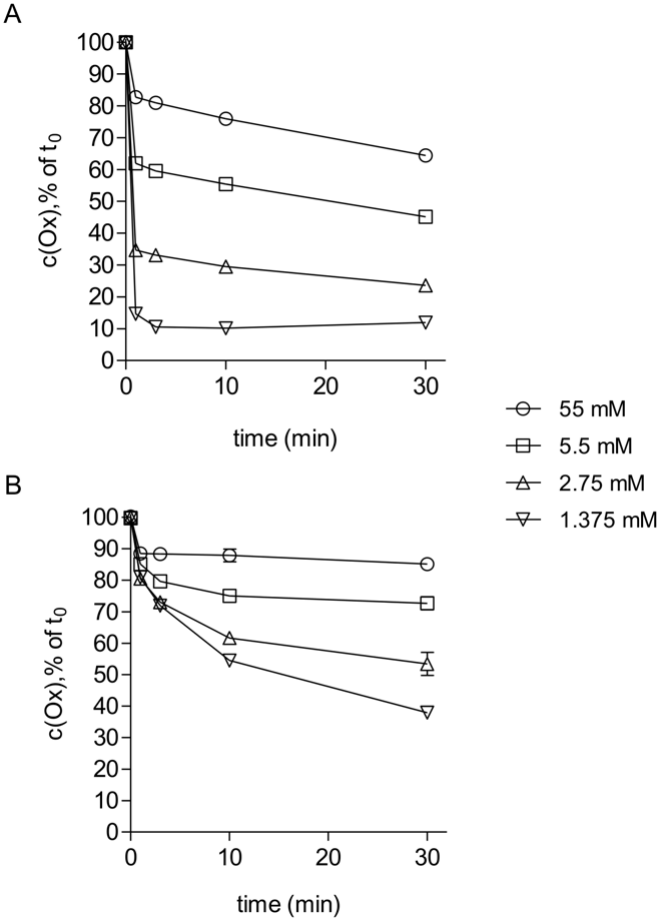
Decrease of oxidation capacity of NCT and NVC-422 in EHEC Direct Medium. Stock solutions of NCT (A) or NVC-422 (B) were 10-fold diluted in this medium to final concentrations of 55 mM, 5.5 mM, 2.75 mM and 1.375 mM. The oxidation capacity was measured with redox potentiometry after indicated time-points. Results with NVC-612 resembled those of NCT. Mean values ± SD from three independent experiments are shown.

To estimate the maximum oxidation capacity available for inactivation of Stx2 in the previous tests, we measured the chlorine consumption of the test oxidants by EHEC Direct Medium using redox-iodometry. Results are depicted in Figure 3A and B. Over a period of 30 min, the oxidation capacity of 55 mM NCT and NVC-612 declined to 64%, of 5.5 mM to 45%, of 2.75 mM to 25.5% and of 1.375 mM to 12% of its basic value. The oxidation capacity of 55 mM NVC-422 decreased to 85%, of 5.5 mM to 72%, of 2.75 mM to 53.5% and of 1.375 mM to 38%. NVC-422 showed approximately 25% less chlorine consumption in EHEC Direct Medium compared to NCT and NVC-612.

### Binding of Stx2 on GEnC Cells (FACS Analysis)

**Figure 4:**
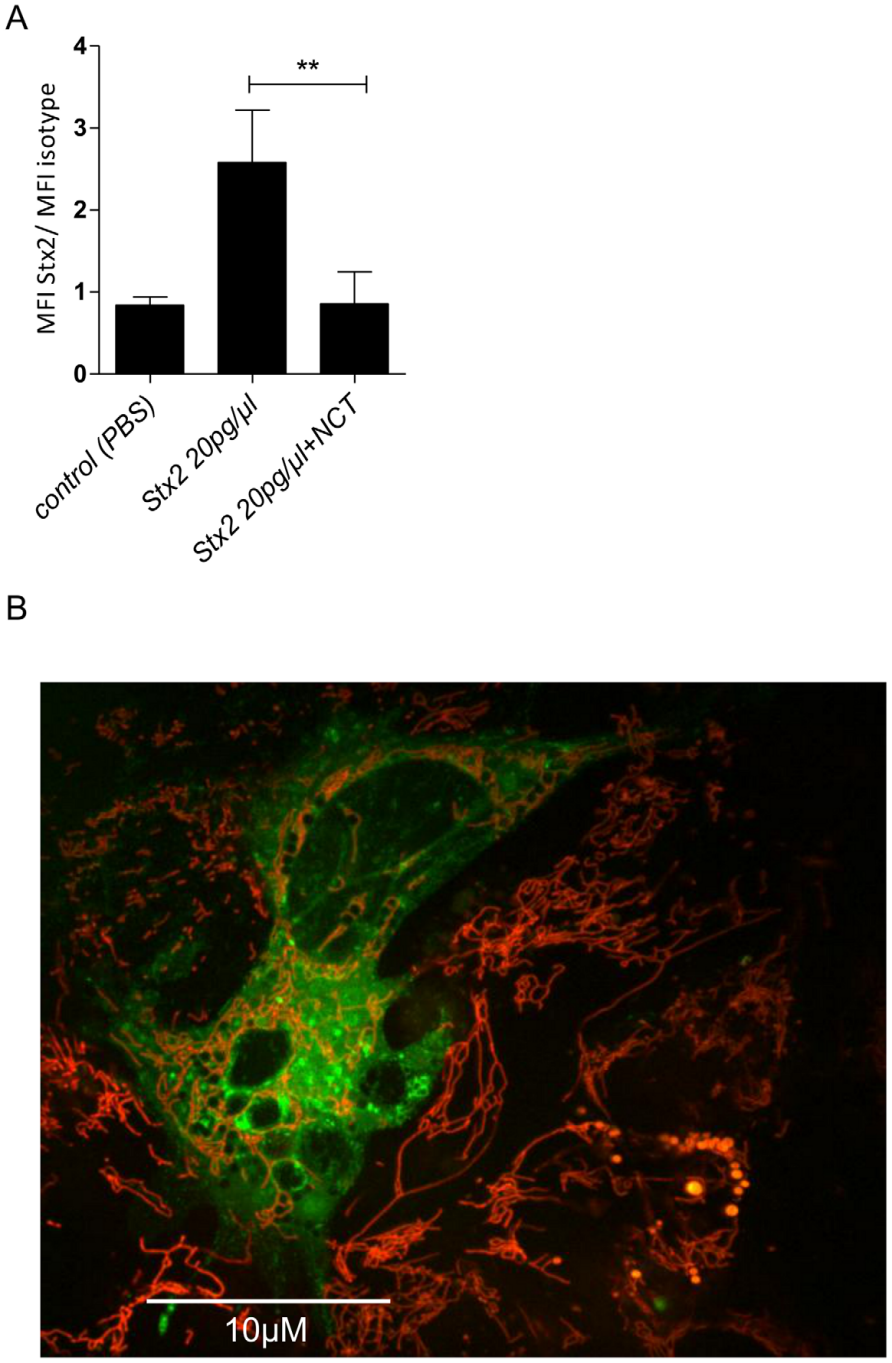
Binding to and penetration of purified Stx2 into human kidney glomerular endothelial cells (GEnC) was inhibited by NCT. (A) FACS analysis of binding of Stx2 to GEnC cells. Purified Stx2 was incubated with 55 mM NCT (1% w/v) in PBS at 37°C for 30 min. Controls with Stx2 in PBS or without Stx2 were performed in parallel. Cells were incubated for 4 h at 37°C. Stx2 bound on the cell surface was detected using a mouse-anti-Stx2-antibody and a FITC-labeled polyclonal goat-anti-mouse secondary antibody. MFI mean fluorescence intensity. Mean values ± SD from three independent experiments are shown. (B) Confocal laser scanning microscopy of penetration of Stx2 into GEnC cells. Labeling of Stx2 was done with Oyster®-488. Subsequently, labeled Stx2 was incubated in 55 mM NCT (1% w/v) or PBS (control) for 30 min at 37°C. The sample was 100-fold diluted in the cell culture, and the fluorescence was monitored. The figure shows a fluorescent cell treated with Stx2 in PBS after 1h (one representative of three independent experiments, 6000× magnification, scale bar 10 µm). By contrast, using NCT-treated Stx2, no fluorescence occurred, indicating the absence of penetration.

After treatment with 55 mM NCT for 30 min, purified Stx2 lost its ability to bind to cell surfaces. Extracellular accumulation of Stx2 was completely absent on cell surfaces when incubated with NCT-treated Stx2 compared to the untreated control (Figure 4A).

### Penetration of Stx2 into GEnC Cells (Confocal Laser Scanning Microscopy)

Confocal laser scanning microscopy revealed no binding or uptake of Stx2 treated with NCT. However, the accumulation and uptake of Stx2 treated with PBS was clearly shown (Figure 4B).

### Structural Changes of Stx2 by NCT Evaluated by SDS-PAGE

**Figure 5:**
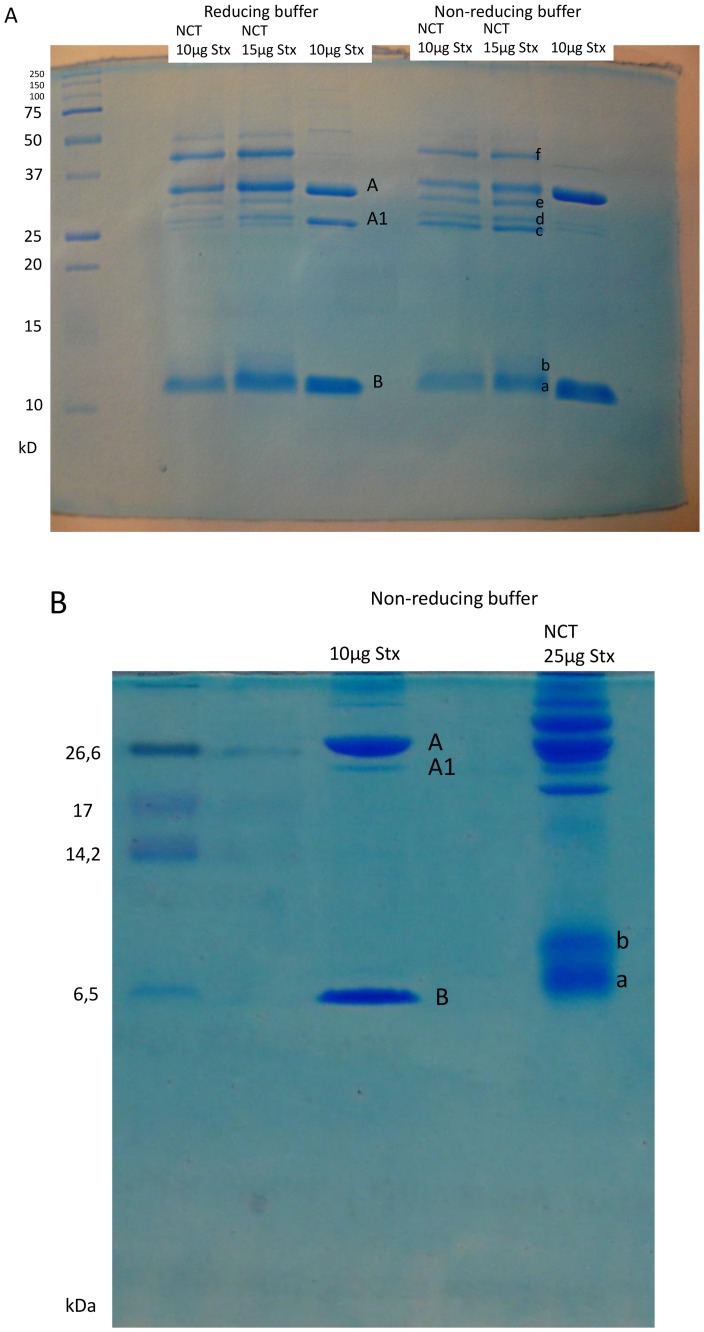
Impact of NCT on purified Stx2 demonstrated in gel electrophoresis. (A) Purified Stx2 was treated with 55 mM NCT for 30 min at RT. Then, an aliquot was used for SDS-PAGE. Reducing buffer contained 4.5% v/v mercaptoethanol. A gel containing bands of aliquots containing 10 or 15 µg Stx2 is shown (one representative of three independent experiments). Bands a–f were subjected to in-gel digestion and mass spectrometry. (B) Purified Stx2 was treated with 55 mM NCT for 30 min at RT and subjected to a tricine gel for improved separation of the low molecular weight bands. Note the separation of the B subunit in bands a and b, which were subjected to in-gel digestion and mass spectrometry.

Coomassie stained gels as shown in Figure 5 revealed the known separation of the purified Stx2 in A and B subunits (lanes 10 µg Stx). Treatment with NCT for 30 min revealed a slight shift of the A subunit band and the B subunit band (Figure 5A). The impact on subunit A1 (to be seen in the reducing buffer) was even more pronounced. In total, six marked new bands occurred with the NCT treated Stx2 in non-reducing buffer (designated a–f in Figure 5), and six in reducing buffer, which indicate fundamental molecular changes of the toxin. Bands a, b, c, d, and e from the sample dissolved in non-reducing buffer were cut out for mass spectrometry analysis to detect also changes that might have been reversed by mercaptoethanol of the reducing buffer. For improved separation of the low molecular weight range, a tricine gel with non-reducing buffer was performed with even higher Stx concentration (25 µg) for the test lane (Figure 5B). Indeed, a clear shift and separation of subunit B could be observed. Bands a and b were evaluated by mass spectrometry.

### Structural Changes of Stx2 by NCT Evaluated by Mass Spectrometry

**Figure 6:**
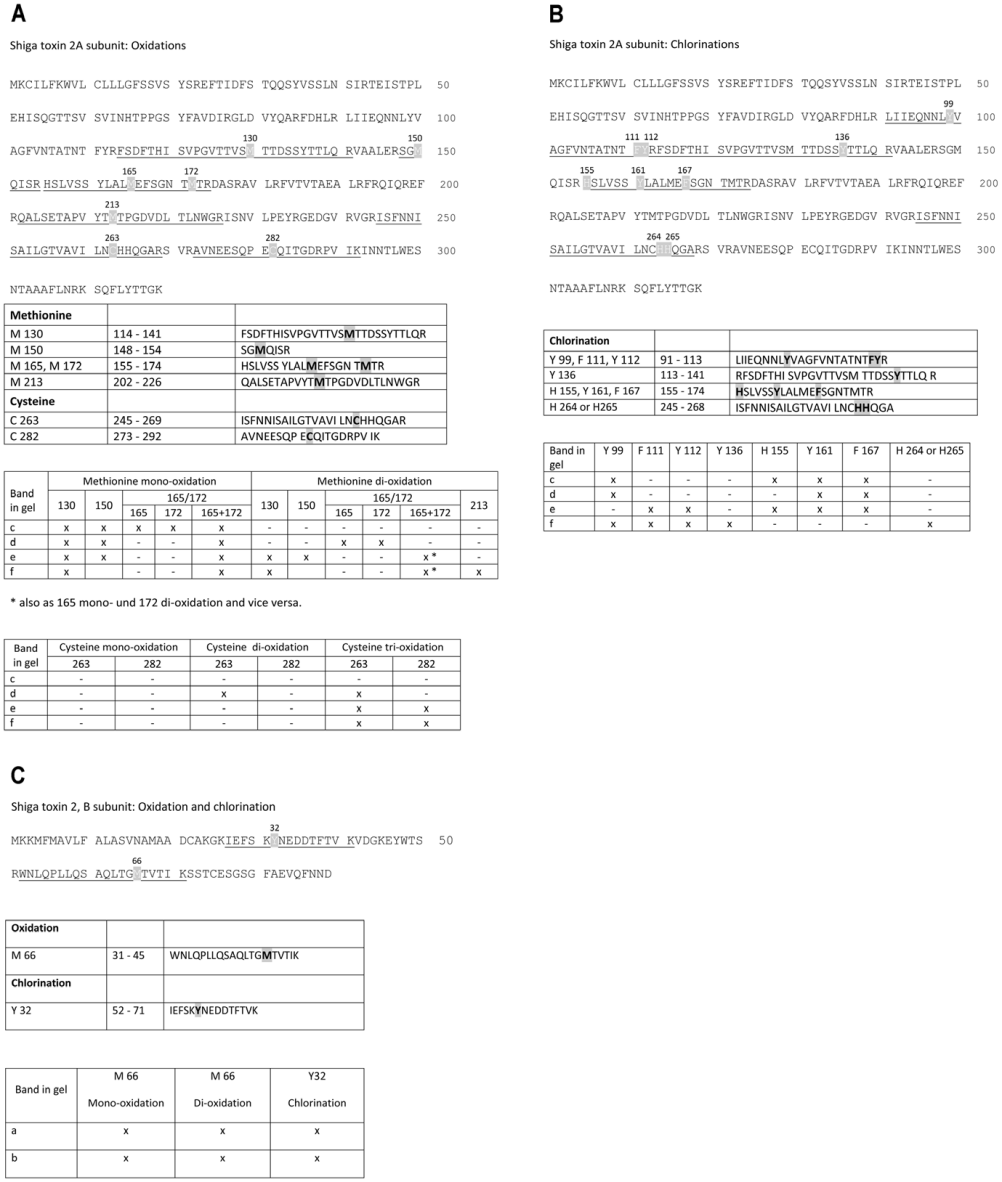
Oxidation of thio groups and aromatic groups of Stx2 detected with mass spectrometry. Bands a-f from the gels, as depicted for one representative in Figure 5, were analyzed with mass spectrometry after in-gel digestion. Similar results gained from three independent experiments are shown. Positions and occurrence in bands are demonstrated. Amino acids 1–22 (subunit A) and 1–19 (subunit B), respectively, comprise the signal peptides. (A) Oxidation of all methionines and cysteines of subunit A occurred. Positions of oxidized amino acids in the sequence are shown and their mono- to tri-oxidation in relation to the bands excised from the gels. (B) Chlorination of phenylalanine, tyrosine, and histidine of subunit A. (C) Oxidation of methionine and chlorination of tyrosine of subunit B.

We were able to show the molecular sites of oxidative attack of subunits A and B of Stx2. Mass spectrometry detected mono- and di-oxidation of methionine. This leads to the formation of sulfoxides (mono-oxidation) and sulfones (di-oxidation). Cysteine showed not only mono- and di-oxidation but also tri-oxidation. In that case sulfenic acid (mono-oxidation), sulfinic acid (di-oxidation) and sulfonic acid (tri-oxidation) were generated. Remarkably, all cysteines and methionines in the subunit A were oxidized. Furthermore, chlorination of the aromatic amino acids phenylalanine, tyrosine, and histidine was found. The exact positions of the oxidized and chlorinated amino acids are illustrated in Figure 6. Notably, in bands a and b from the tricine gel (Figure 5B), we found not only the predominant sequences of subunit B, but also fragments of subunit A (data not shown in Figure 6), indicating oxidative fragmentation of this subunit.

## Discussion

As early as 1918, hypochlorous acid and chloramine T have been shown to remove the lethal effect of *Bacillus welchii* toxin on pigeons after an incubation time of 5 min [26]. Decades later, the active chlorine compounds NaOCl and monochloramine were shown in few studies to inactivate aflatoxins, endotoxins and ricin, mainly in connection with their use as water disinfectants [27], [28], and recently as irrigation solutions for root canals in odontology [29]. The finding that mainly NCT, but also other chloramines are produced by leukocytes as long-lived oxidants with antiinflammatory and antimicrobial properties, implicated their importance for the human defense system [1], [4], [5], [30]. Rapid loss of virulence of pathogens and first studies on inactivation or downregulation of the virulence factors gliotoxin and aspartyl proteinases in the presence of NCT [9], [17]–[19] prompted us to investigate in detail the impact of this substance against a model toxin. In this study we have shown that the even more stable dimethylated analogs NVC-422 and NVC-612 have similar properties as NCT, indicating importance not only for innate immunity, but also for the application of these substances as topical antiinfectives in medicine.

The first step of the investigations, incubation of EHEC in NCT, revealed that inhibition of bacterial growth and Stx2 concentration in the supernatant are tightly connected. Interestingly, this was slightly different with Candida spp. in a previous study, where a decrease of aspartyl proteinases was found by NCT concentrations too low to inhibit fungal growth [18]. Therefore, the minimal NCT-concentration to inhibit virulence factors in relation to the minimal inhibitory concentration seems to be specific for each factor. It is known that the first attack of NCT leads to chlorination of the surface of pathogens (“chlorine cover”, [16]). This impact is sufficient to induce a loss of virulence, as demonstrated in the mouse peritonitis model for *S. aureus* and *S. pyogenes*, but not a loss of viability of the bacteria [9], [17], [18]. Therefore, it is near ad hand that virulence factors on the surface of pathogens and secreted ones will be oxidized and may be inactivated. This is confirmed by the direct inactivation of Stx2 by NCT in the present study and of gliotoxin in a recent one [19]. For killing of bacteria and fungi, penetration of NCT is necessary [9], [16], [31], and it is logical that in this case also virulence factors will be inactivated keeping in mind the multiple sites of attack in *E. coli* found by proteomics [31]. However, at sublethal concentrations of NCT (see Figure 1) it remains unclear if minimal amounts of active chlorine can penetrate bacteria or if signal transduction plays a role. Additionally, it is an open question whether toxin production or release or both are inhibited under these conditions. Also the inhibition of bacterial growth by NCT per se may contribute significantly to the absence of toxin in the supernatant.

The direct chemical impact of NCT on the toxin could be clearly proved by SDS-PAGE and mass spectrometry. N-chloro amino acids are known to react with thio groups (sulfhydryls and thioethers), aromatic C-H compounds, and amino groups [11]. Oxidation capacity is lost by the first two kinds of reaction, while the latter one leads to transfer of the active chlorine to the reaction partner (transchlorination, transhalogenation, [1], [11]). In perfect accordance, we found oxidation of cysteines, methionines, and the aromatic amino acids histidine, phenylalanine, and tyrosine in the subunit A of Stx2. Transchlorination could not be detected in our setup, which is well explainable by the instability of the formed chloramines during the preparation process including gel electrophoresis and in-gel digestion. As a consequence of these chemical reactions, gel electrophoresis with NCT-treated Stx2 revealed bands with lower, but also higher molecular weight (Figure 5). The latter phenomenon has recently been observed with the toxin ricin, lactate dehydrogenase, and lysozyme treated with hypochlorous acid or (to a lesser extent) with monochloramine and has been explained by formation of higher-order aggregates [27]. Moreover, ovalbumin-chlorination by hypochlorous acid has been found to promote formation of aggregates stabilized by non-covalent bonds [32]. Although a definite explanation has not yet been provided, it is clear that both fragmentation and aggregation of target proteins by NCT and other active chlorine compounds result from oxidation of the described groups.

Cystine, tyrosine, and methionine are present in active sites of Stx2 [21]. Since we found oxidation of all cysteines and methionines and chlorination of tyrosine 99 in subunit A, inactivation of the toxin by NCT was a logic consequence and clearly proven in the Vero cell assays. Notably, cysteines 263 and 282 form the disulfide bond between subunit A1 and A2 [21], so that their oxidation will probably lead to conformational changes.

It has to be taken into account that a part of the oxidation capacity is rapidly consumed by thio groups of the medium in which the toxin was dissolved (decline within 1 min, see Figure 3), and that this part relatively increases if the applied concentration of NCT is lowered. Therefore, the remaining free concentration of NCT to inactivate Stx2 was lower than the added one. From these tests can be deduced that approximately 1–3 mM NCT is the threshold of antitoxic activity. The consumption of the dichloro compound NVC-422 was approximately 25% lower than that of NCT and NVC-612. Although, in accordance to that, NVC-422 showed a higher antimicrobial activity, its minimal antitoxic concentration was similar. The reason for that is not known in detail, but the observation fits to previous data that dichloro representatives of active chlorine compounds are not in all cases more active than the monochloro ones [33]. A comparison between Vero cell assays and EHEC growth curves reveals that a higher concentration of NCT (about 3 mM) is needed for inactivation of Stx2 than for growth inhibition (about 2 mM).

**Figure 7:**
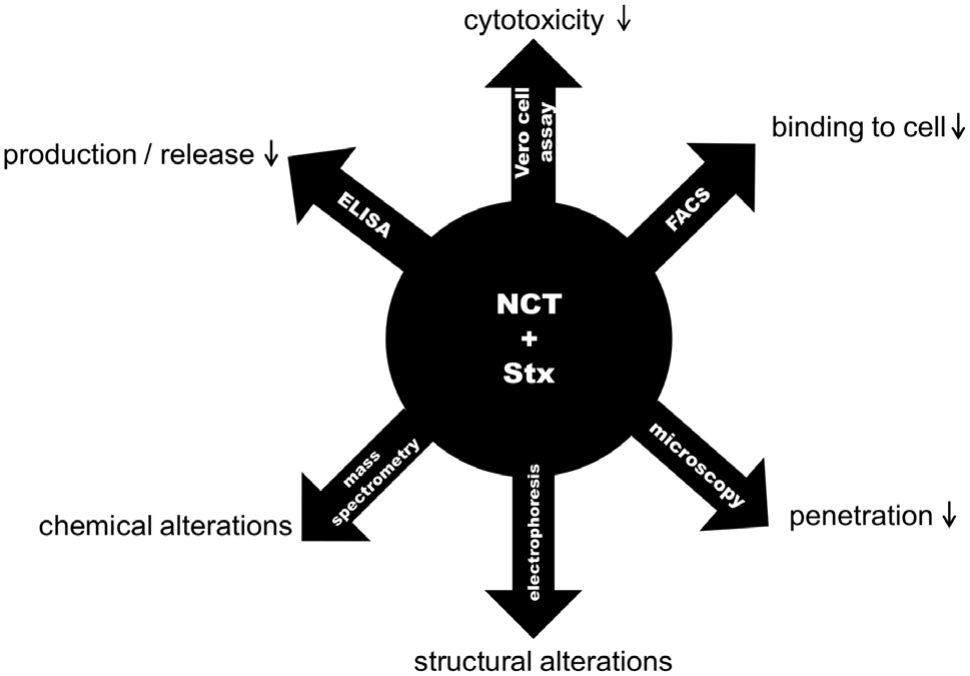
Survey of the impacts of NCT on Stx2. The found effects of NCT on Stx2 are summarized in this figure.

In subunit B of Stx2, we found oxidation of methionine and chlorination of tyrosine, too. This perfectly fits to FACS analysis and confocal microscopy, which demonstrated that NCT treated Stx2 loses its capability of binding and penetrating its target cell, which are functions of the subunit B. From these tests and from the impact found on subunit A, it can be deduced that the toxin is oxidized at multiple sites, including those decisive for its activity (Figure 7). Such a multi-target attack is typical for active chlorine compounds and obviously connected with the broad spectrum of antimicrobial activity and the absence of resistance development of pathogens [9], [11], [12], [31].

One may consider also the other way around that NCT might oxidize the body cells and impact the Stx receptor CD77 (globotriaosylceramide), thus preventing penetration of the toxin. However, since NCT incubated with Stx was at least largely inactivated by dilution before addition to the kidney cells (Figure 4) and since NCT does not react with amides such as CD77 (as opposed to amines [11]), it must be assumed that indeed the impact on the toxin was causative for the found absence of penetration of NCT-treated toxin and not an impact on the receptor.

Antitoxic activity of long-lived oxidants is one more aspect of their antimicrobial activity and might be of importance for both their role in innate immunity and their application for local treatment of infections. Regarding innate immunity, these compounds are created via myeloperoxidase at the sites of infection – as a mixture of different chloramines, from which NCT is a major component [1], [2]. The question is, if the concentration of long-lived oxidants would be sufficient in vivo to inactivate microbes or their toxins. In the supernatant of stimulated granulocytes, up to approximately 100 µM have been found [1], [2], [9], and also within the phagocytic granules the concentrations of chlorine compounds are generally estimated as sufficient to inactivate or at least attack bacteria [34], [35]. Moreover, *in vivo*, chloramine levels of up to 288±96 µM were measured in sputum samples of cystic fibrosis patients [36]. Taking into account chlorine consumption in sputum, rather high levels can be conceived to be produced by leukocytes. It is also assumed that long-lived oxidants may accumulate during inflammation and persist for h in view of permanently arriving granulocytes [5]. Because of all these results, it may be conceived that chloramines are also involved in oxidation of products of pathogens produced *in vivo*. Neutrophilic infiltration has been found in glomeruli of patients with hemolytic uremic syndrome [23], [24]. Generally, such infiltration connected with complement activation is thought to trigger the tissue destruction [37]. However, in view of our results, there might be also beneficial effects like inactivation of Stx, which would fit to the goal of leukocyte migration to remove a nox in the body.

Upon application of NCT for topical treatment of infections, sufficient concentrations to inactivate bacterial toxins are achieved with high probability. In different body regions, for instance crural ulcers, the eye, the outer ear, the oral cavity, and the urinary tract, 55 mM (1% w/v) NCT has been well tolerated and effective in clinical trials [12]. This concentration does not only kill different kinds of microbes after incubation times of a few min to 4 h [12], but also removed the virulence of staphylococci and streptococci within 1 min proven in the mouse peritonitis model [9], [17], and impaired secretory aspartyl proteinases of *Candida albicans* before viability decreased [18]. Since we clearly demonstrated multiple oxidation of the example virulence factor Stx2 and its rapid loss of function within 1–5 min by 55 mM NCT in addition to these findings, it is very probable that not only the microorganisms, but also their products are inactivated during topical treatment. The analogs NVC-612 and NVC-422 obviously have similar properties with the advantage of higher stability at room temperature.

## Materials and Methods

### Chemicals, Test Solutions, and Media

Pure NCT as a crystalline sodium salt (mol wt 181.57 g/mol) was prepared according to [11]. Purity was proved by spectrophotometry [11]. Pure NVC-612 (molecular weight 209.63 g/mol) and NVC-422 (molecular weight 244.07 g/mol) were from NovaBay Pharmaceuticals Inc. (Emeryville, CA, USA). Fresh solutions of 55 mM (equals 1% w/v NCT) or 550 mM (equals 10% w/v NCT) of each of these test substances were prepared in 0.1 M phosphate buffer at pH 7.1 and diluted in this buffer to the final concentrations as appropriate. Mueller-Hinton agar was from Merck, fetal calf serum from Invitrogen (10% v/v in RPMI), RPMI-1640 Medium from Sigma and EHEC Direct Medium 1055 rp from Heipha (Heipha Dr. Müller GmbH, Eppelheim, Germany). The EHEC Direct Medium was used to grow EHEC. In contrast to conventional media, it contains 50 ng/ml mitomycin C, which stimulates the Stx production. EBM-2 medium with growth supplemental kit was from LONZA Cologne AG (Köln, Germany). Purified Stx2 (5 mg/ml), a kind gift from Helge Karch (Münster, Germany), was used to perform gel electrophoresis and mass spectrometry, FACS analysis and confocal microscopy [38].

### Growth of EHEC and Stx2 Production in the Presence of Sublethal Concentrations of NCT

Clinical isolates EHEC 178 and EHEC 22 (both O157:H7, Stx1 negative, positive for Stx2, *eae*-coding intimin, and serine protease EspP), all deep frozen for storage, were grown on Mueller-Hinton agar plates and subsequently in EHEC Direct Medium at an agitation of 160/min and 37°C overnight to 3–5×10^8^ colony forming units (CFU)/ml. Stock solutions of NCT and NVC-612 (55 mM each) were diluted to 16.5 mM, 22 mM, and 27.5 mM in 0.1 M phosphate buffer, NVC-422 to 5.5 mM, 11 mM, and 16.5 mM. Bacterial suspensions (40 µl) were mixed with 3560 µl EHEC Direct Medium and 400 µl of the prepared test solutions, respectively. After 0, 2, 4, and 6 h at 37°C, aliquots of 200 µl were removed, and the half (100 µl) from these was diluted 100-fold in 0.9% w/v NaCl. Immediately afterwards, aliquots (50 µl) of these dilutions were spread in duplicate on tryptic soy agar plates with an automatic spiral plater (model WASP 2, Don Whitley Scientific, Shipley, UK), allowing a detection limit of 100 CFU/ml taking into account both plates and the dilution. Addition of inactivation substances for the used oxidants was not necessary under these test conditions as tested in a pilot experiment. The plates were incubated at 37°C, and colony forming units of microorganisms were counted after 24 and 48 h. To determine the presence of Stx2 in the samples, the other half of the aliquots (100 µl) of these dilutions were subjected to Stx Premier ELISA Kit (Meridian Bioscience, Inc. Cincinnati, OH, USA) in accordance to the instructions of the manufacturer.

### Influence of NCT on the Cytotoxic Effect of Stx2 against Vero Cells

Vero cells (kidney monkey cells, ATCC CCL-81) were grown at 37°C and 5% v/v CO_2_ in RPMI 1640 medium plus 10% v/v fetal calf serum in cell culture flasks. Subcultures were grown in 96 well microtitre plates to a density of approximately 90% confluency. EHEC 178 and 22 were grown in EHEC Direct Medium overnight as described above. After centrifugation, the supernatants were sterile filtered (0.45 µm, Sartorius). Solutions of 550 mM, 55 mM, 27.5 mM and 13.75 mM NCT, NVC-422, and NVC-612 each were prepared in 0.1 M phosphate puffer (pH 7.1). 900 µl of filtered supernatants were mixed with 100 µl of test solutions (NCT, NVC-422, or NVC-612) and incubated for 30 min. Additional experiments were done with 55 mM NCT and incubation times of 1, 5, 10, 15, 20, 25, and 30 min. Subsequently, samples were 100-fold diluted in RPMI + 10% v/v FCS to decrease the concentration of N-chloro amino acids under the toxic level for cells (toxic threshold>0.55 mM NCT in RPMI + 10% v/v FCS with unlimited incubation,>5.5 mM NCT in RPMI + 10% v/v FCS or even in non-chlorine-consuming buffers for at least 60 min incubation). 100 µl each of these diluted samples were given to the cells in 100 µl RPMI + FCS. After 72 h, cells that showed the typical cytopathic effect caused by Stx2 were counted at a 100-fold magnification. To confirm these results, cell viability was evaluated by trypan blue exclusion test and release of lactate dehydrogenase (Cytotoxicity Detection Kit LDH, Roche, Mannheim, Germany) in accordance to the instructions of the manufacturer. Controls without oxidants, without EHEC supernatant, with taurine or dimethyltaurine and supernatant, with plain RPMI + FCS, and with supernatant of *E. coli* ATCC 11229, a Shiga toxin negative strain, were performed in parallel. Igepal CA-630 1% (Sigma), which lyses the cells, was used as a positive control for the LDH assay.

### Consumption of Oxidation Capacity by EHEC Medium

Since active chlorine compounds react with organic residues of media such as the used EHEC Direct Medium [11], [12], oxidative activity is consumed in part (so-called chlorine consumption). This effect has to be taken into account for correct interpretation of the results, if the application of such media can’t be avoided. Therefore, we measured the decrease of oxidation capacity with redox-iodiometry [39]. To gain similar conditions as in the Vero cell assays, 3600 µl of EHEC Direct Medium were mixed with 400 µl of buffered NCT, NVC-422, or NVC-612 solutions to get final concentrations of 55 mM, 5.5 mM, 2.75 mM and 1.375 mM. Subsequently, the redox potential after reaction with iodide was determined using a PHM250 Ion Analyzer (Radiometer, Copenhagen, Denmark), and the remaining oxidation capacity was calculated as described previously [39].

### Binding of NCT-treated Stx2 to GEnC (FACS Analysis)

Human kidney glomerular endothelial cells (GEnC) were a kind gift from Simon Satchell PhD, Academic Renal Unit, University of Bristol [40]. GEnC were cultured in EBM-2 medium and growth factor kit as described [41]. Purified Stx2 was incubated with 55 mM NCT (1% w/v) in PBS at 37°C for 30 min. For FACS analysis, cells were incubated with 20 pg/µl NCT-treated Stx2. Controls with Stx2 in PBS or without Stx2 were performed in parallel. Cells were incubated for 4 h at 37°C. Stx2 bound on the cell surface was measured using a mouse-anti-Stx2-antibody purchased from Hycult biotech (Uden, Netherlands). Secondary antibody was FITC-labeled polyclonal goat-anti-mouse (DAKO, Glostrup, Denmark). Antibody dilutions were chosen as given in the manufacturers' protocol for FACS. Cell viability was checked with propidium iodide assay (Sigma-Aldrich).

Cells were prepared in standard procedure for FACS analysis. Measurement of 5.000 events out of 150.000 cells per sample was carried out by BD FACS Canto II. Mouse IgG1:FITC (GM4992), ordered from invitrogen, was used as isotype control. Results were given as ratio of mean fluorescence intensity (MFI) of sample and MFI of isotype to avoid biases due to background fluorescence.

### Penetration of NCT-treated Stx2 into GEnC (Confocal Laser Scanning Microscopy)

Labeling of Stx2 was done with Oyster®-488 Antibody Labeling Kit (Luminartis, Muenster, Germany) as described by the manufacturer. Subsequently, labeled Stx2 was incubated in 55 mM NCT (1% w/v) in PBS (or plain PBS for controls) for 30 min at 37°C. Then, an aliquot of 2 µl of this mixture was added to the cells in 200 µl cell culture medium to reveal a final concentration of 20 pg/µl of labeled Stx2 and to reduce the concentration of NCT below cytotoxic levels. Immediate confocal laser scanning microscopy to visualize the intracellular accumulation of labeled Stx2 was performed on a microlens-enhanced Nipkow disk-based confocal system UltraVIEW RS (Perkin Elmer, Wellesley, USA) mounted on an inverted microscope (IX-70, Olympus, Nagano, Japan). Images were taken using the UltraVIEW RS software (Perkin Elmer) in 6000x magnification with oil.

To exclude destruction of Oyster® 488-TFP by NCT, both components were mixed at the same concentrations and conditions as for confocal laser scanning microscopy. Immediately afterwards, 10 µl were pipetted onto an object slide without additives and monitored by fluorescence microscopy over a period of 30 min. In addition, 1 µl of Oyster® 488-TFP was 100-fold diluted with 10% w/v lysine in water followed by a 10-fold dilution in 1% w/v NCT or saline control. The fluorescence signal was only minimally decreased compared to the fluorescent dye without NCT in both settings, so that the tests were not biased by a reaction of NCT with the dye.

### Structural Changes of Stx2 by NCT Evaluated by SDS-polyacrylamid Gel Electrophoresis (SDS-PAGE)

An electrophoresis system from Biorad was used to perform SDS-PAGE with a 16% gel. To exceed the minimum of 5µg protein required for mass spectrometry, we used 10 µg and 15 µg Stx2 per slot. To achieve these quantities of Stx2, 4 µl purified Stx2 (5 µg/µl) were mixed with 20 µl 1.2% w/v NCT (dissolved in phosphate buffer, pH 7.1), and additionally 6 µl purified Stx2 with 18 µl 1.2% w/v NCT, and incubated for 30 min at RT. A control was performed in parallel without NCT. The samples were bisected into 12 µl each and mixed with 3 µl reducing or non-reducing buffer each. The sample buffer consisted of 250 µl 20% w/v SDS, 500 µl 0.5 M Tris buffer, 200 µl glycerol and 100 µl 1% w/v bromphenol blue solution.The reducing buffer contained 50 µl mercaptoethanol as a reducing substance (final concentration 4.5% w/v). Subsequently all samples were boiled at 95°C for 5 min and placed on ice immediately afterwards.

For improved separation of low molecular-weight proteins, particularly for Stx2 subunit B, we applied a 16.5% tricine gel containing 6M urea, as described by Schägger [42]. Gels were stained with coomassie blue. For mass spectrometry, only bands from samples in non-reducing buffer (without mercaptoethanol) were cut out to detect oxidations of thiols.

### Structural Changes of Stx2 by NCT Evaluated by Mass Spectrometry

The in-gel digestion was performed as published previously [43]. Protein digests were analyzed using an UltiMate 3000 nano-HPLC system (Dionex, Germering, Germany) coupled to an LTQ Orbitrap XL mass spectrometer (ThermoScientific, Bremen, Germany) equipped with a nanospray ionization source. A homemade fritless fused silica nano-capillary column (75 µm i.d. × 280 µm o.d.) packed with 10 cm of 3 µm reversed-phase C_18_ material (Reprosil) was used. The gradient (solvent A: 0.1% v/v formic acid; solvent B: 0.1% v/v formic acid in 85% v/v acetonitrile) started at 4% B. The portion of solvent B was increased linearly from 4% to 50% during 50 min and from 50% to 100% during 5 min. A flowrate of 250 nL/min was applied. Database search was performed in Proteome Discoverer (version 1.2.0.208, ThermoScientific) using the Sequest search engine and a database consisting of 545 sequences to which the sequences of Shiga toxin 2, A and B subunit, were added. Variable modifications were oxidation and di-oxidation at Cys and Met, tri-oxidation at Cys, and chlorination at Phe, His, Trp, and Tyr. Specific cleavage sites for trypsin (KR) were selected with two missed cleavage sites allowed. Peptide tolerance was±10 ppm and MS/MS tolerance was±0.8 Da. The criteria for positive identification of peptides were Xcorr >2.3 for doubly charged ions, and Xcorr >2.8 for triply charged ions.

For MS analysis, the displayed sequence (Figure 6) and the corresponding numbers were taken from the up-to-date UniProt database (including the signal peptide) with the accession numbers Q8XBV2 for Stx2, subunit A, and Q8X531 for Stx2, subunit B. The signal peptide comprised amino acids 1–22 for subunit A and 1–19 for subunit B.

### Statistics

Data are presented as mean values and standard deviations (SD). Student’s unpaired *t* test in case of two groups, or one-way ANOVA and Bonferroni’s and Dunnett’s multiple comparison test in case of more than two groups were used to test for a difference between the test and control group. P<0.05 was considered significant for all tests. Calculations were done with GraphPad Prism 5 software.

## Supporting Information

Figure S1 Inhibition of growth of EHEC 22 and of Stx2 production in the presence of sublethal concentrations of NCT and NVC-422. (A) CFU counts of EHEC in Direct Medium, to which NCT was added to final concentrations of 0 mM (control), 1.65 mM, 2.2 mM and 2.75 mM at 37°C. (B) Stx2 produced by EHEC under the same conditions as in (A), measured by ELISA, related to the 6 h value of the control without NCT. (C) CFU counts of EHEC in Direct Medium, to which NVC-422 was added to final concentrations of 0 mM (control), 0.55 mM, 1.1 mM and 1.65 mM at 37°C. (D) Stx2 produced by EHEC under the same conditions as in (C), measured by ELISA, related to the 6 h value of the control without NVC-422. Mean values ± SD from three independent experiments are shown in (A-D). *P < 0.05; **P < 0.01.
(TIF)

## References

[1] Grisham MB, Jefferson MM, Melton DF, Thomas EL (1984) Chlorination of endogenous amines by isolated neutrophils. J Biol Chem 259: 10404–10413. 6381484

[2] Weiss SJ, Klein R, Slivka A, Wei M (1982) Chlorination of taurine by human neutrophils. J Clin Investig 70: 598–607. 6286728 10.1172/JCI110652 PMC370261

[3] Weiss SJ (1989) Tissue destruction by neutrophils. N Engl J Med 320: 365–376. 2536474 10.1056/NEJM198902093200606

[4] Koprowski M, Marcinkiewicz J (2002) Taurine chloramine - its role in immunity and new perspectives for clinical use. Centr Eur J Immunol 27: 69–74.

[5] Marcinkiewicz J (1997) Neutrophil chloramines: missing links between innate and acquired immunity. Immunol Today 18: 577–580. 9425735 10.1016/s0167-5699(97)01161-4

[6] Park E, Jia J, Quinn MR, Schuller-Levis G (2002) Taurine chloramine inhibits lymphocyte proliferation and decreases cytokine production in activated human leukocytes. Clin Immunol 102: 179–184. 11846460 10.1006/clim.2001.5160

[7] Kanayama A, Inoue J, Sugita-Konishi Y, Shimizu M, Miyamoto Y (2002) Oxidation of Ikappa Balpha at methionine 45 is one cause of taurine chloramine-induced inhibition of NF-kappa B activation. J Biol Chem 277: 24049–24056. 11983684 10.1074/jbc.M110832200

[8] Marcinkiewicz J, Walczewska M, Olszanecki R, Bobek M, Biedron R, et al (2009) Taurine haloamines and heme oxygenase-1 cooperate in the regulation of inflammation and attenuation of oxidative stress. Adv Exp Med Biol 643: 439–450. 19239176 10.1007/978-0-387-75681-3_46

[9] Nagl M, Hess M, Pfaller K, Hengster P, Gottardi W (2000) Bactericidal activity of micromolar N-chlorotaurine - evidence for its antimicrobial function in the human defence system. Antimicrob Agents Chemother 44: 2507–2513. 10952603 10.1128/aac.44.9.2507-2513.2000 PMC90093

[10] Passo SA, Weiss SJ (1984) Oxidative mechanisms utilized by human neutrophils to destroy *Escherichia coli* . Blood 63: 1361–1368. 6326895

[11] Gottardi W, Nagl M (2002) Chemical properties of N-chlorotaurine sodium, a key compound in the human defence system. Arch Pharm Pharm Med Chem 335: 411–421. 10.1002/1521-4184(200212)335:9<411::AID-ARDP411>3.0.CO;2-D 12447914

[12] Gottardi W, Nagl M (2010) N-chlorotaurine, a natural antiseptic with outstanding tolerability. J Antimicrob Chemother 65: 399–409. 20053689 10.1093/jac/dkp466

[13] Iovino SM, Krantz KD, Blanco DM, Fernandez JA, Ocampo NF, et al (2011) NVC-422 topical gel for the treatment of impetigo. Int J Clin Exp Pathol 4: 587–595. 21904634 PMC3160610

[14] Wang L, Khosrovi B, Najafi R (2008) *N*-Chloro-2, 2-dimethyltaurines: a new class of remarkably stable *N*-chlorotaurines. Tetrahedron Lett 49: 2193–2195.

[15] Wang L, Belisle B, Bassiri M, Xu P, Debabov D, et al (2011) Chemical Characterization and Biological Properties of NVC-422, a Novel, Stable N-Chlorotaurine Analog. Antimicrob Agents Chemother 55: 2688–2692. 21422212 10.1128/AAC.00158-11 PMC3101424

[16] Gottardi W, Nagl M (2005) Chlorine covers on living bacteria: the initial step in antimicrobial action of active chlorine compounds. J Antimicrob Chemother 55: 475–482. 15761074 10.1093/jac/dki054

[17] Nagl M, Hengster P, Semenitz E, Gottardi W (1999) The postantibiotic effect of N-chlorotaurine on *Staphylococcus aureus.* Application in the mouse peritonitis model. J Antimicrob Chemother 43: 805–809. 10404319 10.1093/jac/43.6.805

[18] Nagl M, Gruber A, Fuchs A, Lell C, Lemberger EM, et al (2002) Impact of N-chlorotaurine on viability and production of secreted aspartyl proteinases of *Candida* spp. Antimicrob Agents Chemother 46: 1996–1999. 12019124 10.1128/AAC.46.6.1996-1999.2002 PMC127226

[19] Reeves EP, Nagl M, O'Keeffe J, Kelly J, Kavanagh K (2006) Effect of N-chlorotaurine on Aspergillus, with particular reference to destruction of secreted gliotoxin. J Med Microbiol 55: 913–918. 16772419 10.1099/jmm.0.46405-0

[20] Orth D, Würzner R (2010) Complement in typical hemolytic uremic syndrome. Semin Thromb Hemost 36: 620–624. 20865638 10.1055/s-0030-1262883

[21] Fraser ME, Fujinaga M, Cherney MM, Melton-Celsa AR, Twiddy EM, et al (2004) Structure of shiga toxin type 2 (Stx2) from Escherichia coli O157:H7. J Biol Chem 279: 27511–27517. 15075327 10.1074/jbc.M401939200

[22] Orth D, Khan AB, Naim A, Grif K, Brockmeyer J, et al (2009) Shiga toxin activates complement and binds factor H: evidence for an active role of complement in hemolytic uremic syndrome. J Immunol 182: 6394–6400. 19414792 10.4049/jimmunol.0900151

[23] Sauter KA, Melton-Celsa AR, Larkin K, Troxell ML, O'Brien AD, et al (2008) Mouse model of hemolytic-uremic syndrome caused by endotoxin-free Shiga toxin 2 (Stx2) and protection from lethal outcome by anti-Stx2 antibody. Infect Immun 76: 4469–4478. 18694970 10.1128/IAI.00592-08 PMC2546846

[24] Inward CD, Howie AJ, Fitzpatrick MM, Rafaat F, Milford DV, et al (1997) Renal histopathology in fatal cases of diarrhoea-associated haemolytic uraemic syndrome. British Association for Paediatric Nephrology. Pediatr Nephrol 11: 556–559. 9323279 10.1007/s004670050337

[25] Exeni RA, Fernandez GC, Palermo MS (2007) Role of polymorphonuclear leukocytes in the pathophysiology of typical hemolytic uremic syndrome. ScientificWorldJournal 7: 1155–1164. 17694250 10.1100/tsw.2007.172 PMC5901139

[26] Taylor HD, Austin JH (1918) The action of antiseptics on the toxin of Bacillus welchii: a preliminary note. J Exp Med 27: 375–381. 19868211 10.1084/jem.27.3.375 PMC2125964

[27] Cole KD, Gaigalas A, Almeida JL (2008) Process monitoring the inactivation of ricin and model proteins by disinfectants using fluorescence and biological activity. Biotechnol Prog 24: 784–791. 18386939 10.1021/bp070362b

[28] Yang CY (1972) Comparative studies on the detoxification of aflatoxins by sodium hypochlorite and commercial bleaches. Appl Microbiol 24: 885–890. 4631102 10.1128/am.24.6.885-890.1972 PMC380691

[29] Maekawa LE, Valera MC, Oliveira LD, Carvalho CA, Koga-Ito CY, et al (2011) In vitro evaluation of the action of irrigating solutions associated with intracanal medications on Escherichia coli and its endotoxin in root canals. J Appl Oral Sci 19: 106–112. 21552710 10.1590/S1678-77572011000200005 PMC4243747

[30] Zgliczynski JM, Stelmaszynska T, Domanski J, Ostrowski W (1971) Chloramines as intermediates of oxidation reaction of amino acids by myeloperoxidase. Biochim Biophys Acta 235: 419–424. 4378090 10.1016/0005-2744(71)90281-6

[31] Arnitz R, Sarg B, Ott HW, Neher A, Lindner H, et al (2006) Protein sites of attack of N-chlorotaurine in Escherichia coli. Proteomics 6: 865–869. 16372277 10.1002/pmic.200500054

[32] Olszowski S, Olszowska E, Stelmaszynska T, Krawczyk A, Marcinkiewicz J, et al (1996) Oxidative modification of ovalbumin. Acta Biochim Pol 43: 661–672. 9104502

[33] Gottardi W, Hagleitner M, Nagl M (2005) N,N-Dichlorotaurine: chemical and bactericidal properties. Arch Pharm Pharm Med Chem 338: 473–483. 10.1002/ardp.200500146 16211659

[34] Klebanoff SJ (2005) Myeloperoxidase: friend and foe. J Leukoc Biol 77: 598–625. 15689384 10.1189/jlb.1204697

[35] Winterbourn CC, Hampton MB, Livesey JH, Kettle AJ (2006) Modeling the reactions of superoxide and myeloperoxidase in the neutrophil phagosome: implications for microbial killing. J Biol Chem 281: 39860–39869. 17074761 10.1074/jbc.M605898200

[36] Witko Sarsat V, Delacourt C, Rabier D, Bardet J, Nguyen AT, et al (1995) Neutrophil-derived long-lived oxidants in cystic fibrosis sputum. Am J Respir Crit Care Med 152: 1910–1916. 8520754 10.1164/ajrccm.152.6.8520754

[37] Orth-Höller D, Riedl M, Würzner R (2011) Inhibition of terminal complement activation in severe Shiga toxin-associated HUS - perfect example for a fast track from bench to bedside. EMBO Mol Med 3: 617–619. 21954211 10.1002/emmm.201100169 PMC3377111

[38] Zhang W, Bielaszewska M, Pulz M, Becker K, Friedrich AW, et al (2008) New immuno-PCR assay for detection of low concentrations of shiga toxin 2 and its variants. J Clin Microbiol 46: 1292–1297. 18272709 10.1128/JCM.02271-07 PMC2292924

[39] Gottardi W, Pfleiderer J (2005) Redox-iodometry: a new potentiometric method. Anal Bioanal Chem 382: 1328–1338. 15981007 10.1007/s00216-005-3247-8

[40] Satchell SC, Anderson KL, Mathieson PW (2004) Angiopoietin 1 and vascular endothelial growth factor modulate human glomerular endothelial cell barrier properties. J Am Soc Nephrol 15: 566–574. 14978158 10.1097/01.asn.0000115397.22519.03

[41] Satchell SC, Tasman CH, Singh A, Ni L, Geelen J, et al (2006) Conditionally immortalized human glomerular endothelial cells expressing fenestrations in response to VEGF. Kidney Int 69: 1633–1640. 16557232 10.1038/sj.ki.5000277

[42] Schagger H, von JG (1987) Tricine-sodium dodecyl sulfate-polyacrylamide gel electrophoresis for the separation of proteins in the range from 1 to 100 kDa. Anal Biochem 166: 368–379. 2449095 10.1016/0003-2697(87)90587-2

[43] Sobieszek A, Matusovsky OS, Permyakova TV, Sarg B, Lindner H, et al (2006) Phosphorylation of myorod (catchin) by kinases tightly associated to molluscan and vertebrate smooth muscle myosins. Arch Biochem Biophys 454: 197–205. 16970905 10.1016/j.abb.2006.08.004

